# Immobilization of Homogeneous Monomeric, Oligomeric and Fibrillar Aβ Species for Reliable SPR Measurements

**DOI:** 10.1371/journal.pone.0089490

**Published:** 2014-03-03

**Authors:** Daniel Frenzel, Julian M. Glück, Oleksandr Brener, Filipp Oesterhelt, Luitgard Nagel-Steger, Dieter Willbold

**Affiliations:** 1 Forschungszentrum Jülich, ICS-6 Structural Biochemistry, Jülich, Germany; 2 Heinrich-Heine-Universität Düsseldorf, Institut für Physikalische Biologie, Düsseldorf, Germany; University of Oldenburg, Germany

## Abstract

There is strong evidence that the amyloid-beta peptide (Aβ) plays a central role in the pathogenesis of Alzheimer's disease (AD). In this context, a detailed quantitative description of the interactions with different Aβ species is essential for characterization of physiological and artificial ligands. However, the high aggregation propensity of Aβ in concert with its susceptibility to structural changes due to even slight changes in solution conditions has impeded surface plasmon resonance (SPR) studies with homogeneous Aβ conformer species. Here, we have adapted the experimental procedures to state-of-the-art techniques and established novel approaches to reliably overcome the aforementioned challenges. We show that the application of density gradient centrifugation (DGC) for sample purification and the use of a single chain variable fragment (scFv) of a monoclonal antibody directed against the amino-terminus of Aβ allows reliable SPR measurements and quality control of the immobilized Aβ aggregate species at any step throughout the experiment.

## Introduction

Alzheimer's disease (AD) is the most common form of neurodegenerative disorders. While several genetic risk factors have been identified to be associated with the onset of AD, the major risk factor of AD is age. In 2010 around 35 million people were affected worldwide. With increasing life expectancy, >65 million cases are predicted by 2030 [1]. One of the hallmarks of AD is the appearance of amyloid aggregates [2]. According to the amyloid cascade hypothesis there is evidence that cellular events leading to cell death in AD are initiated by different amyloid beta-peptide (Aβ) assembly states [3]. This is supported by the observation of extracellular amyloid-like aggregates – mainly consisting of Aβ [4] – in the central nervous system of patients suffering from AD. Formation of Aβ is catalyzed by proteolytic cleavage of the amyloid precursor protein (APP) by β-secretase and γ-secretase [5]. The role of the oligomeric and fibrillar assembly states in disease progression is still debatable.

Nevertheless, due to the strong evidence that Aβ plays a central role in the pathogenesis of AD, substantial efforts aim to develop assays that, on the one hand, either allow the detection and quantification of Aβ species in biological matrices as for instance cerebrospinal fluids [6], [7] or brain tissue [8]–[10] or, on the other hand, allow the characterization of compounds that target different Aβ species and/or interfere with their formation. A critical measure describing such compounds is their binding affinities to distinct Aβ species. Thus, reliable assays are urgently needed for quantitative affinity determination between ligands and the various Aβ species.

An optimal assay for binding studies with Aβ-binding molecules should combine minimal consumption of label-free ligands with maximum yield on kinetic and thermodynamic binding data. The surface plasmon resonance (SPR) technology can meet these requirements. In an SPR experiment, one of the interactants is immobilized (ligand) on a sensor chip surface. With regard to the propensity of Aβ(1–42) to aggregate, its use as the ligand is a clear advantage as stable fixation minimizes the risk of structural rearrangements, oligomerization and aggregation throughout the experiment.

However, several challenges exist with respect to the design of an SPR-assay for Aβ: (i) For obtaining robust data it is a prerequisite to use preparation techniques that ensure reliable preparation of homogeneous Aβ species that are free from undesired aggregation states. Aβ readily forms different oligomeric species that vary in size. Hence, samples are frequently heterogeneous, which prevents satisfactory data evaluation and binding curve fitting even when using multi-compound binding models. (ii) It is essential to find a suitable immobilization technique that is compatible with the buffer where formation of the respective Aβ species was performed because this prevents structural rearrangement of the prepared species. Many studies have analyzed the effect of different buffer components, temperature and pH on the formation of different Aβ species [11]. Taken together even slight changes in one of the latter physical parameters can cause structural rearrangements and peptide instability. Unfortunately, the majority of immobilization techniques in SPR are based on chemical reactions that require a change of solution conditions. Moreover, immobilization of variable amounts of ligand on the surface in a highly reproducible manner is another critical step as this allows adaption of the Rmax (maximum response obtainable when all available ligand binding places are occupied) to the molecular weight of the interaction partner. Since there is a linear dependence of molecular mass to the detected SPR response, analytes with high molecular weights yield higher response signals than smaller analytes [12]. Excessive amounts of ligand eventually lead to heterogeneity in recorded sensorgrams during binding experiments, hampering data evaluation because of mass transport limitations and rebinding events. Generally, the lower the amount of immobilized ligand that yields a sufficient response during interaction with an analyte the lower the risk of heterogeneity. (iii) In addition to preparation and immobilization, special requirements for the measurement procedure are also present. A standard SPR assay includes regeneration steps between multiple analyte injections. This introduces the risk of critical structural rearrangements in the immobilized Aβ aggregates. It would therefore be beneficial to circumvent this step to yield reproducible interaction data. (iv) A reference molecule for quality control purposes of the surface activity during every single step of an experiment would greatly facilitate experimental design. Accordingly, selection of a proper analyte for assay development and surface characterization is crucial. It should be available in sufficient amounts, possess an on- and off-rate within SPR instrument specifications without introducing avidity effects.

## Methods

### Expression of the single chain variable fragment IC16

For recombinant production of scFv-IC16, *E. coli* BL21 DE3 pRARE2 was transformed with the expression vector pET22b-scFv-IC16-5His. Each 1 l 2YT (10 g l-1 yeast extract, 20 g l-1 tryptone, 10 g l-1 NaCl, 10 ml l-1 20% dextrose, 5 ml l-1 2 M MgCl_2_, chloramphenicol and ampicillin, pH 7.4) expression culture was inoculated with an aliquot of a 50 ml overnight LB (5 g l-1 yeast extract, 10 g l-1 tryptone, 10 g l-1 NaCl) culture (grown at 37°C, 150 rpm) to a final OD_600_ of ∼0.1. Cells were grown at 37°C (150 rpm) to an OD_600_ of 1.6–1.8. Subsequently, cultures were chilled for 1 hour at 4°C until IPTG was added to a final concentration of 0.2 mM for induction of scFv-IC16 protein expression. Expression was carried out for 24 hours at 18°C under gentle agitation (150 rpm). Cells were harvested by centrifugation (30 min, 4°C, 3750 rpm), pellets washed with PBS (10 mM sodium phosphate buffer pH 7.4, 137 mM NaCl, 2.7 mM KCl) and stored at −20°C until further use.

### Purification of scFv-IC16

Pellets were resuspended in 20 ml lysis buffer I (50 mM Tris-HCl pH 8.0, 1 mM EDTA, 1 mg/ml lysozyme), supplemented with protease inhibitors (Complete EDTA-free Protease Inhibitor Cocktail Tablets, Roche). For cell lysis 20% Triton X-100 was added to a final concentration of 1%. MgCl_2_ was added to a final concentration of 20 mM together with 500 U DNAse I. After an incubation at RT for ∼15 minutes the volume was adjusted to 50 ml with lysis buffer II (8.33 mM imidazole, 833 mM NaCl, 16.6 mM CaCl_2_, 1% Triton X-100) followed by centrifugation for 30 min at 20,000 g. Pellets containing scFv-IC16 in inclusion bodies were resuspended in 30 ml binding buffer (50 mM Tris-HCl pH 7.8, 500 mM NaCl, 8 M urea) followed by overnight incubation at 4°C in an orbital shaker. Suspensions were centrifuged (45 min, 20,000 g) and supernatants containing scFv-IC16 were purified by denaturing Ni^2+^-NTA-chromatography. Affinity chromatography was performed with Ni^2+^-loaded nitrilotriacetic acid (NTA) agarose from Qiagen (column volume, CV, of 3 ml) that was equilibrated with binding buffer. Supernatant was loaded onto the column by gravity flow, followed by washing steps with two CVs of wash buffer I (50 mM Tris-HCl pH 6.0, 500 mM NaCl, 8 M urea) and two CVs of wash buffer II (50 mM Tris-HCl pH 5.3, 500 mM NaCl, 8 M urea). scFv-IC16 was eluted with elution buffer (50 mM Tris-HCl pH 4.0, 500 mM NaCl, 8 M urea). All fractions were analyzed by SDS-PAGE with subsequent Coomassie staining and scFv-IC16-containing fractions were pooled. For refolding, renaturation buffer (50 mM Tris-HCl, 500 mM NaCl, 1% Triton X100, pH 7.2) was added to elution fractions in a 10∶1 ratio (v/v). Afterwards, a second affinity chromatography purification was performed with Aβ(1–16) coupled NHS-sepharose (Pierce). After equilibration with a 10∶1 mixture of refolding and elution buffer fractions containing scFv-IC16 were loaded onto the column. A washing step with 10 CVs TBS (50 mM Tris-HCl, 150 mM NaCl, pH 7.4) removed non-bound material. Elution was achieved with 50 mM glycine, pH 2.5. Each elution fraction was immediately neutralized by addition of 50 µl 2 M Tris-HCl, pH 8.0 per ml fraction volume and checked by SDS-PAGE. Fractions containing scFv-IC16 were pooled, dialyzed against PBS, and concentrated to a final concentration of 5 µM with Vivaspin 20 columns from Sartorius Stedim (3000 MWCO PES).

### Preparation of Aβ(1–42) monomers and oligomers by size exclusion chromatography (SEC)

The protocol used by Johansson, Berglind-Dehlin, Karlsson, Edwards, Gellerfors and Lannfelt [13] was adapted for Aβ(1–42) monomer and oligomer preparation by SEC with minor modifications. Lyophilized stocks of Aβ(1–42) (Bachem), carboxy-terminally biotinylated Aβ(1–42) (Eurogentec) and amino-terminally biotinylated Aβ(1–42) (Anaspec) were separately dissolved in 100% hexafluoroisopropanol (HFIP) and incubated overnight at RT. In the case of oligomer preparations, amino-terminally biotinylated Aβ(1–42) and non-biotinylated Aβ(1–42) were mixed in a 1∶10 ratio. After incubation, solutions were divided into 125 µg aliquots. HFIP was removed by evaporation in a Concentrator 5301 (Eppendorf). Aβ(1–42) was resolubilized in 100 µl SEC-buffer (50 mM sodium phosphate buffer, 150 mM NaCl, 0.6% Tween 20, pH 7.4) and briefly centrifuged (30 s) at 15,000 *g* to sediment insoluble material immediately prior to separation by SEC. Separation was performed with a Superdex 75 10/300 GL column operated at RT by an Äkta purifier system at a flow rate of 0.8 ml min^−1^. For each single run ∼100 µl of solubilized Aβ(1–42) was loaded onto the column. Monomers eluted at ∼14 ml, whereas oligomers eluted close to the void volume (at ∼8 ml). Samples were immediately used for immobilization on sensor chip surfaces. Initially, for establishment of the immobilization assay a BCA-assay was used to correlate the absorbance at 214 nm of the SEC fractions to the overall Aβ concentration. We observed, that an A_214_ = 250 mAU (oligomers) or A_214_ = 150 mAU (monomers) in the size exclusion chromatogram correlates with ∼1 µM total Aβ concentration derived from a BCA-assay. Omitting the BCA-assay step dramatically reduces the time between elution and immobilization. For immobilization of monomers and oligomers (10% amino-terminally biotinylated) Aβ(1–42) concentrations of ∼10 nM and ∼100 nM, respectively, were used.

### Preparation of Aβ(1–42) fibrils

The protocols used by Wood, Maleeff, Hart and Wetzel [14] and Nagel-Steger, Demeler, Meyer-Zaika, Hochdorffer, Schrader and Willbold [15] were adapted for Aβ(1–42) fibril preparation with minor modifications. Lyophilized aliquots of Aβ(1–42) and amino-terminally biotinylated Aβ(1–42) were dissolved separately in 100% HFIP and incubated at RT overnight. Biotinylated Aβ(1–42) and non-biotinylated Aβ(1–42) in HFIP were mixed in a 1∶10 ratio and subsequently divided into 72 µg aliquots. HFIP was removed by evaporation in a Concentrator 5301 (Eppendorf). Then, Aβ pellets were solubilized in 200 µl sodium phosphate buffer pH 7.4 (10 mM; yielding an 80 µM Aβ solution) and incubated for 24 hours at 25°C (600 rpm). To separate the hereby obtained fibrils from smaller aggregates and from monomers, they were subjected to a density gradient centrifugation (DGC) step. The gradient was prepared in thin-walled ultracentrifugation tubes (Ultra-Clear 11×34 mm, 2.2 ml from Beckman) by successively overlaying the following volumes of density gradient solutions: 260 µl 50%, 260 µl 40%, 260 µl 30%, 780 µl 20%, 260 µl 10% and 100 µl 5% (v/v) Iodixanol in 10 mM sodium phosphate buffer pH 7.4. After addition of 100 µl sample, the gradient was centrifuged (3 h, 4°C, 259,000 *g*) using a TLS-55 rotor in a benchtop ultracentrifuge Optima TL-100 (Beckman-Coulter). From each gradient 14 fractions of 140 µl were harvested from top to bottom. Fibrils were typically located in fractions 11 to 13.

### Surface Plasmon Resonance (SPR)

For SPR experiments Series S Sensor Chips SA (GE Healthcare Life Sciences) in combination with a Biacore T200 system were used. Series S Sensor Chips SA are coated with streptavidin and allow ligand immobilization based on the biotin-streptavidin interaction. For our experiments PBS (filtered with 0.2 µm, PVDF) was used as running buffer. After docking a new sensor chip, the system was initiated with a “Prime” command and the detector normalized with 70% glycerol (GE Healthcare Life Sciences). All flow cells were activated with three consecutive one minute injections of 1 M NaCl in 50 mM NaOH. For ligand immobilization the flow rate was adjusted to 5 µl min^−1^ in order to minimize sample consumption. After immobilization we let the flow cells stabilize overnight to remove unspecifically bound material and detergence. To do so, we set the flow speed and temperature to 30 µl min^−1^ and 25°C respectively.

For interaction studies the flow speed and temperature were adjusted to 30 µl min^−1^ and 25°C respectively. All interaction studies were performed in the single-cycle kinetic mode [16]. Here, five different analyte concentrations were injected within a single cycle (contact time: 90 s, final dissociation time after the highest concentration: 1800 s) in order of increasing concentration. The applied analyte concentrations for scFv-IC16 were 312.5, 625, 1250, 2500 and 5000 nM. Binding studies with monoclonal antibody 6E10 were performed with concentrations of 0.32, 1.6, 8, 40 and 200 nM. All binding data were double referenced by collecting data in dual-channel mode with an untreated and therefore not Aβ(1–42) containing reference flow cell connected upstream of the flow cell with the respective Aβ(1–42) assembly state and by the subsequent subtraction of a blank buffer injection (1× PBS) from the obtained binding responses.

Double referenced SPR data were evaluated with Biacore T200 Evaluation Software (version 1.0) using the available binding models. Aβ monomer data was fit to a 1∶1 binding model, whereas sensorgrams of Aβ oligomers and fibrils were analyzed with a heterogeneous ligand binding model accounting for two separate ligand sites for analyte binding. Values for bulk refractive index (R_I_) and mass transfer (k_t_) correction were manually set to zero, because double-referencing was applied and low amounts of ligand were immobilized.

## Results

**Figure 1:**
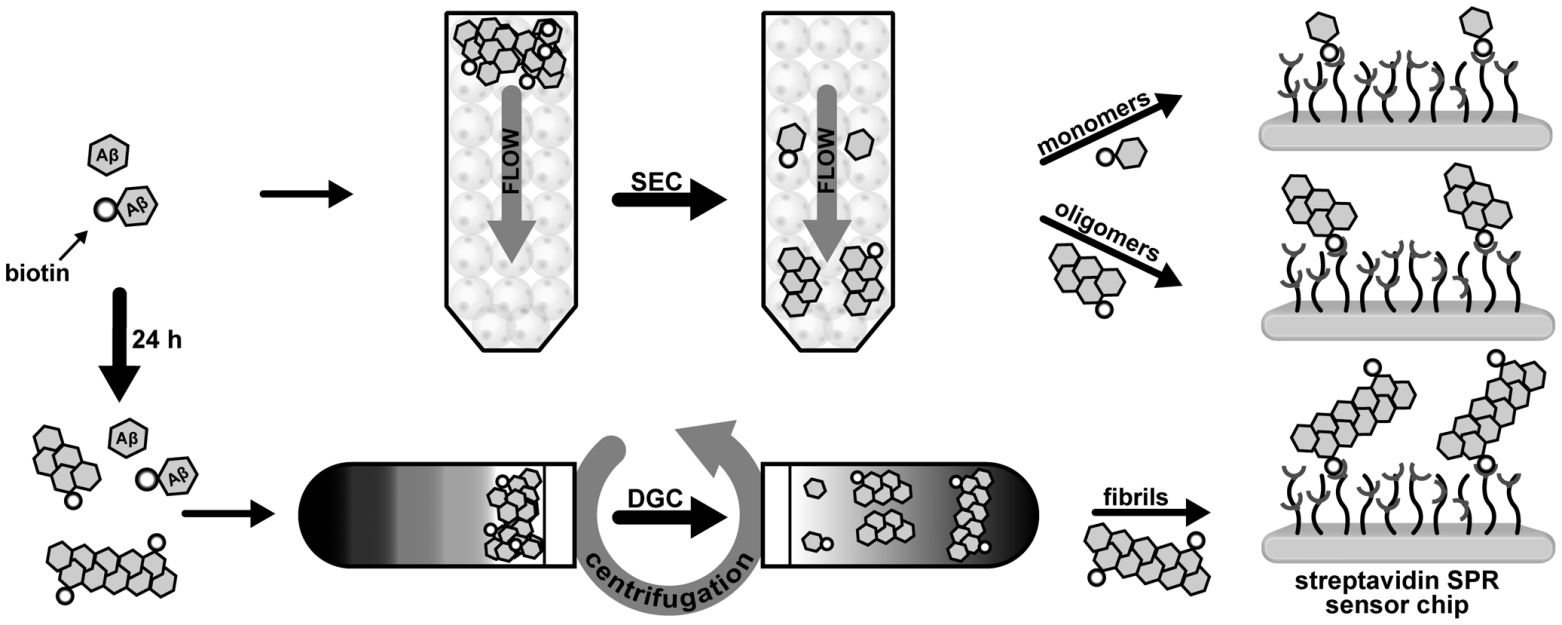
Surface preparation with different Aβ(1–42) assembly states for surface plasmon resonance (SPR) analysis. Size exclusion chromatography (monomers and oligomers) and density gradient centrifugation (fibrils) ensure highly pure samples for immobilization on sensor surfaces and subsequent SPR measurements.

For immobilization of different Aβ(1–42) species we have chosen a streptavidin-biotin-coupling procedure as this avoids change in buffer conditions during Aβ(1–42) immobilization [17] concomitant with many alternative protocols. In addition, due to the strong interaction of streptavidin and biotin with a dissociation constant *K*_D_ of around 10^−15^ M [18] there is virtually no loss of ligand during the experiment. Moreover, streptavidin-biotin-coupling can be used effectively to control the amount of bound ligand simply by varying the concentration of the ligand or the duration of the injection. Fig. 1 shows the experimental setup scheme for the preparation of different Aβ(1–42) species and their immobilization.

Because any harsh regeneration steps between measurements will very likely do harm to the immobilized Aβ(1–42) species, they need to be avoided completely. Instead, very long washing steps have been introduced between separate measurements. To save time, so called kinetic titration [13], where the analyte is injected in increasing concentrations without regeneration steps in-between, is the method of choice. In comparison with classical multi-cycle kinetics, sample consumption and analysis time is reduced and, most importantly, the need for regeneration is eliminated [16], [19]. For analysis of the kinetic titration obtained sensorgrams the tool “single-cycle kinetics” of the Biacore evaluation software package has been used.

For assay development and for quality check of surface characterization of immobilized Aβ species, the single-chain variable fragment (scFv-IC16) of the antibody IC16 that was initially selected to target the first 16 amino-acid residues of Aβ was selected [20], [21]. scFvs are easy to produce and purify, stable at high concentrations for at least weeks, and possess only a single binding site with high specificity for their epitope, thereby avoiding any avidity effects.

### Characterization of Aβ monomers

**Figure 2:**
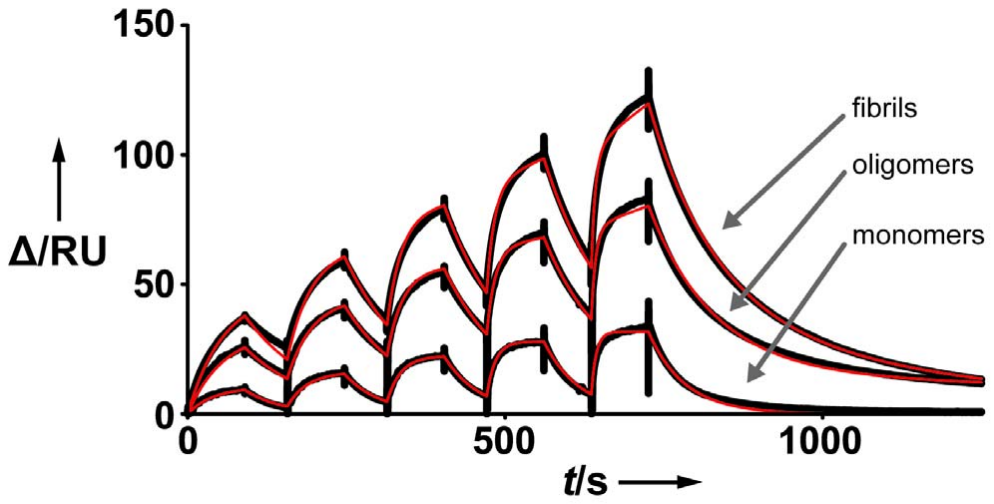
Interaction of scFv-IC16 with different immobilized Aβ(1–42) assembly states. SPR sensorgrams were recorded separately with single-cycle kinetics. Experimentally obtained, double-referenced binding data (black traces) were superimposed with the corresponding fit (red traces). Monomer data was fit to a 1∶1 Langmuir binding model, and oligomer and fibril data were fit to a heterogeneous ligand binding model. ΔRU: delta of the response units. t/s: time in seconds.

To test the specificity of scFv-IC16 to the amino-terminal part of Aβ, the chosen biotin-streptavidin immobilization procedure should be well-suited. The orientation of Aβ on the surface can be modulated easily by changing the location of the biotin tag. In theory it should therefore be possible to hide the epitope of scFv-IC16 by fusion of a biotin tag in close proximity. To do so we have immobilized the amino-terminally biotinylated Aβ(1–42) monomers (Fig. S1 A) that have been purified by size exclusion chromatography (SEC) prior to immobilization (Fig. S2 A) [13]. SEC purification ensures monodispersity of Aβ monomers. Binding of an anti-Aβ(1–42) antibody (6E10) demonstrated that Aβ was successfully immobilized (Fig. S3). In contrast, binding of scFv-IC16 to the N-terminally biotinylated Aβ(1–42) monomer loaded surface could not be detected (for details see Fig. S4). Conversely, C-terminally biotinylated Aβ(1–42) monomers that were immobilized to the surface was bound by both, 6E10 and scFv-IC16 (Fig. 2 and Fig. S5). We conclude that both, N- and C-terminally biotinylated Aβ monomers were successfully immobilized, but immobilization of N-terminally biotinylated Aβ(1–42) on the streptavidin-coated sensor chip restricts binding of scFv-IC16. To extract quantitative information from the experimental data of scFv-IC16 and C-terminally biotinylated Aβ(1–42) we fitted the obtained sensorgrams to a Langmuir 1∶1 binding model. Refractive index correction (R_I_) was not required because all binding data were double referenced prior to analysis. As can be seen in Fig. 2 and Tab. 1, the resulting fit represents the experimental data very well and yields a dissociation constant (*K*_D_) of 0.77 µM. A low χ^2^-value of 4.1 supports this finding. A comparison with steady state affinity analysis (a linear fitting approach, see Fig. S7) revealed an excellent match. With this approach the *K*_D_ determined was equal to 0.97 µM (χ^2^: 0.38).

**Table 1:** Overview of kinetic rates for scFv-IC16 binding to different Aβ(1–42) assembly states obtained within the single-cycle kinetic SPR experiments.

	monomers	monomers#	oligomers#	fibrils#
*k*_a1_^[a]^	2.27*10^4^	2.16*10^4^	2.66*10^4^ (4.9*10^3^; n = 4)	2.96*10^4^ (3.9*10^3^; n = 3)
*k*_d1_^[b]^	1.74*10^−2^	2.03*10^−2^	0.98*10^−2^ (1.8*10^−3^; n = 4)	0.92*10^−2^ (1.5*10^−3^; n = 3)
*K*_D1_^[c]^	7.69*10^−7^	9.36*10^−7^	3.70*10^−7^ (1.4*10^−7^; n = 4)	3.12*10^−7^ (7.5*10^−8^; n = 3)
*k*_a2_^[a]^	-	4.80*10^4^	1.03*10^2^ (9.2*10^1^; n = 4)	4.54*10^2^ (3.7*10^2^; n = 3)
*k*_d2_^[b]^	-	4.78*10^−9^	5.76*10^−4^ (2.9*10^−3^; n = 4)	1.93*10^−3^ (1.0*10^−3^; n = 3)
*K*_D2_^[c]^	-	9.90*10^−14^	5.60*10^−6^ (7.9*10^−6^; n = 4)	4.26*10^−6^ (2.2*10^−6^; n = 3)
?^2^	4.1	2.4	3.0	1.2
*R*_max1_^[d]^	36.6	36.2	69.1	89.3
*R*_max2_^[d]^	-	1.6	206.1	143.1

The hash (^#^) denotes that kinetic rates were determined with a heterogeneous binding model. Standard deviation with number of experiments is given in brackets.

# fit to a heterogeneous binding model. Units are: [a] Ms^−1^, [b] s^−1^, [c] M, and [d] RU.

### Characterization of an example for Aβ oligomers

Next we immobilized Aβ(1–42) oligomers containing 10% amino-terminally biotinylated Aβ(1–42) and checked for successful immobilization of Aβ with 6E10 and scFv-IC16. To reduce the risk of sample heterogeneity we removed species different than oligomers by SEC [13] and immediately immobilized these oligomers (Fig. S1 A). 6E10 and scFv-IC16 were both able to bind to the Aβ oligomers on the surface (see Fig. 2 and Fig. S6). Importantly, since amino-terminally biotinylated monomers are not recognized by scFv-IC16 we can conclude that the obtained responses rely on scFv-IC16 binding to oligomers. Therefore, scFv-IC16 proves to be a powerful tool for surface characterization and quality control of immobilized Aβ and application as a molecular tool for SPR studies with Aβ in higher aggregation states is conceivable. Antibodies and their respective Fab fragments are often known to recognize both, linear and conformational epitopes. Binding curves were therefore fit with a heterogeneous ligand binding model. Again, owing to double referencing, a refractive index (RI) correction was not required. We obtained two separate *K*_D_ values (0.37 µM and 5.60 µM; χ^2^: 3.0) with affinities differing by one order of magnitude. Use of simpler models increased χ^2^ by at least a factor of ten and supports the initial assumption of a second epitope of Aβ(1–42) oligomers for scFv-IC16. This effect is very likely not being caused by rebinding effects of the analyte. The total mass of immobilized Aβ(1–42) oligomers is very comparable with the amount of immobilized Aβ(1–42) monomers, in which clearly no rebinding could be observed (Fig. S1 A).

### Characterization of Aβ fibrils

For immobilization of fibrils, an Aβ(1–42) mixture with 10% amino-terminally biotinylated Aβ(1–42) was used. To ensure the absence of lower molecular weight species we applied density gradient centrifugation (DGC) for separation of fibrils from other oligomeric states and monomers. Iodixanol was used as gradient media because this reagent has several advantages over other potential agents: it is non-ionic, forms self-generated gradients in comparatively short centrifugation times and, most importantly, it is iso-osmotic [22]. This ensures a low influence on protein stability and structure. Nevertheless, to analyze the potential influence of Iodixanol on the structural assembly of the prepared fibrils AFM studies were performed (Fig. S8). The obtained AFM results indicate that fibril formation is not altered by Iodixanol, that the fibrils are virtually identical to Aβ fibrils previously studied by AFM [23] and that no background signal by lower molecular weight species such as oligomers can be observed. As observed for SEC-purified Aβ-oligomers, it was possible to immobilize reproducible amounts of the DGC separated Aβ-fibrils on the surface (Fig. S1 B). To the best of our knowledge, this is the first report on the immobilization of Iodixanol DGC-separated Aβ(1–42) fibrils via a biotin-streptavidin technique and acts as a proof-of-principle experiment that demonstrates this combination of methods as a powerful tool for future sample preparation of ligands for SPR studies. However, the refractive index shows a dramatic jump following sample injection because of the presence of Iodixanol (Fig. S1 B). Incubation of the flow cell in a continuous flow mode revealed a linear decay of 4 RU (RU: response units) per hour after 8 h. We assumed this decay is caused by a small amount of the fibrils dissociating, because similar decays have been observed previously for immobilized fibrils [24]. scFv-IC16 was able to bind to the fibril surface. The binding curves were fit with the identical model used for Aβ(1–42) oligomers. The resulting dissociation constants for scFv-IC16 binding to Aβ(1–42) fibrils were determined to be 0.31 µM and 4.26 µM (χ^2^: 1.2, Tab. 1), which are very similar to those obtained for Aβ oligomers.

## Discussion

**Figure 3:**
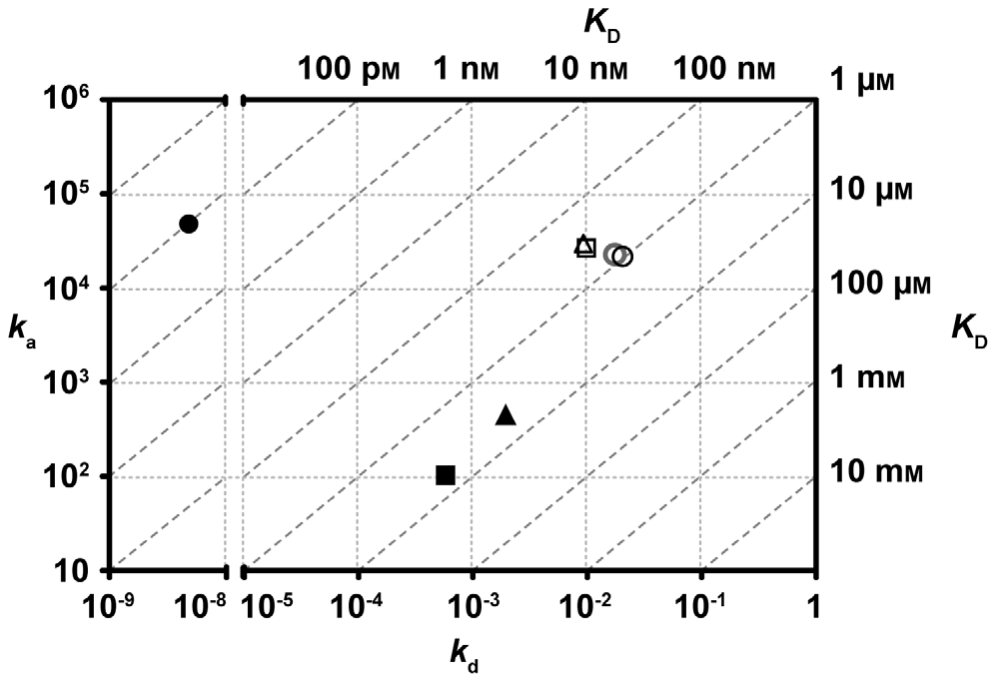
Kinetic rates obtained for scFv-IC16 binding to different immobilized Aβ(1–42) assembly states. Association rate constants (*k*_a_) were plotted against dissociation rate constants (*k*_d_). The dissociation constant (*K*_D_) can be extracted from the diagonal lines. Circles, squares and triangles correspond to data from interactions with monomers, oligomers and fibrils, respectively, whereas filled symbols represent data for the second binding site. All data was determined with the heterogeneous fitting model. The grey circle represents monomer data obtained with a 1∶1 binding model.

A direct comparison of the obtained kinetic rates and overall affinities for scFv-IC16 and Aβ species reveals that for each Aβ assembly state (C-terminally biotinylated monomers, as well as 10% N-terminally biotinylated oligomers and fibrils), there is one interaction component present with nearly identical properties among all three assembly states (Fig. 3. and Tab. 1). The attained association and dissociation rates for the high affinity site of scFv-IC16 binding to Aβ monomers, oligomers and fibrils are 2.3×10^4^ Ms^−1^ and 1.7×10^−2^ s^−1^, 2.7×10^4^ Ms^−1^ and 1.0×10^−2^ s^−1^, 3.0×10^4^ Ms^−1^ and 0.9×10^−2^ s^−1^, respectively. Based on these rate constants, it is tempting to speculate that the same binding epitope for scFv-IC16 is present in each of the studied Aβ assembly states. Because this epitope is obviously missing in purely N-terminally biotinylated monomers, we can conclude that this epitope contains the very N-terminal residues of Aβ. Moreover, the affinity of the slower binding reaction of scFv-IC16 binding oligomers and fibrils was nearly one order of magnitude weaker (K_D2_-values in Tab. 1). Based on this observation we conclude that Aβ generates a secondary binding site for scFv-IC16 when forming higher assembly states like oligomer and fibril structures. Remarkably, fitting of sensorgram data obtained with scFv-IC16 binding monomeric Aβ(1–42) to the heterogeneous ligand binding model, as used for oligomers and fibrils, did not yield a second binding component similar to the oligomer and fibril data. Instead, an unlikely apparent *K*_D_ of 9.9×10^−14^ M in concert with an *R*_max_ value of 1.6 supports the notion that scFv-IC16 binding data for monomers follows a 1∶1 Langmuir interaction, which confirms that the Aβ monomer preparation was extremely homogeneous, and that the secondary binding epitope existing in oligomers and fibrils is clearly not a fitting artefact.

In addition to the specific findings concerning the properties of scFv-IC16, we hereby describe a general approach to immobilize any Aβ assembly that contains a fraction of n-terminally biotinylated Aβ molecules to streptavidin-coated SPR chips while having the possibility to confirm integrity of the immobilized Aβ species via binding of scFv-IC16 at any time of the experiment. IC16 was chosen, because it recognizes the amino-terminus of Aβ only, when it is not biotinylated and bound to streptavidin. The scFv fragment of IC16 has been chosen because it binds in a 1∶1 ratio to the target and is eluting more rapidly than the full-length IC16 antibody. The setup allows immobilization of either C-terminally Aβ monomers or any kind of higher order Aβ forms that were artificially prepared employing a fraction of N-terminally biotinylated Aβ. We have given one example each of a monomer, oligomer and fibril preparation, as were published previously by others.

## Conclusions

Taken together, we have established a novel approach allowing reproducible interaction studies with different homogeneous Aβ(1–42) assembly states by SPR. SEC and DGC purification of Aβ species prior to streptavidin-biotin coupling ensures sample homogeneity and minimal surface alterations, which are major limitations of SPR experiments involving Aβ. In addition, we have employed a monoclonal antibody-derived scFv for direct verification of successfully immobilized higher Aβ assembly states. Although, the hereby described approach is straightforward only for in vitro-generated Aβ assemblies, it may prove to be an essential step toward future screening and in-depth characterization of potential drug candidates and thereby has the capability to greatly simplify and accelerate drug development for AD.

## Supporting Information

Figure S1 **Overlay of sensorgrams obtained during immobilization of three different Aβ(1–42) assembly states on a streptavidin sensor chip.** Oligomers and fibrils were prepared in a 1∶10 molar ratio of amino-terminally biotinylated and non-biotinylated Aβ(1–42), whereas monomers were completely biotinylated at the carboxy-termini. Final immobilized amounts are given in brackets. Shown are examples of sensorgrams obtained during immobilization of Aβ(1–42) monomers and oligomers (A) as well as fibrils (B). Because the procedure involves changes in buffer, these sensorgrams don't allow conclusions about association and dissociation rates of the immobilized Aβ(1–42) assembly states. After a few hours a stable baseline decay dependent on the immobilized assembly state was reached. RU: response units.
(PNG)

Figure S2 **Size exclusion chromatography profile at 214 nm of A) 100% C-terminal biotinylated Aβ(1–42), B) 100% N-terminal biotinylated Aβ(1–42), C) 10% N-biotinylated Aβ(1–42)/90% Aβ(1–42), D) Molecular weight standard with Aprotinin (6.5 kDa), Lysozyme (14.4 kDa) and Conalbumin, Catalase, Aldolase, Ferritin in the void volume with Superdex 75 10/300 GL.** Oligomers elute partly within the void volume and the monomers at ∼9 kDa. AU: absorption units at 214 nm.
(PNG)

Figure S3 **SPR sensorgram depicting binding of monoclonal IgG antibody 6E10 to N-terminally biotinylated Aβ(1–42) monomers immobilized on a streptavidin-coated SPR sensor chip.**
(PNG)

Figure S4 **SPR sensorgrams depicting binding pattern of scFv IC16 to ∼1200 RU N-terminally biotinylated Aβ(1–42) monomers, immobilized on a streptavidin-coated SPR sensor chip.**
(PNG)

Figure S5 **SPR sensorgram depicting binding of monoclonal IgG antibody 6E10 to C-terminally biotinylated Aβ(1–42) monomers immobilized on a streptavidin-coated SPR sensor chip.**
(PNG)

Figure S6 **SPR sensorgram depicting binding of monoclonal IgG antibody 6E10 to Aβ(1–42) oligomers immobilized on a streptavidin-coated SPR sensor chip.** Aβ oligomers were composed of a 1∶10 ratio of amino-terminally biotinylated Aβ(1–42) and non-biotinylated Aβ(1–42).
(PNG)

Figure S7 **Steady-state analysis of scFv-IC16 binding to immobilized C-terminally biotinylated Aβ(1–42) monomers.** The dissociation constant *K*_D_ for a 1∶1 interaction is calculated from equation R_eq_ = (C*R_max_)/(*K*_D_+C), where C refers to the analyte concentration, R_eq_ to the obtained equilibrium binding levels and R_max_ to the maximum analyte binding capacity of the surface. Values for *K*_D_ and R_max_ were determined to 0.97 µM and 39.7 RU with a corresponding χ^2^ value of 0.38.
(PNG)

Figure S8 **Analysis of Aβ(1–42) fibrils after density gradient centrifugation (DGC) by atomic force microscopy.** The fibrils were created in 10 mM sodium phosphate buffer (pH 7.4) and separated by density gradient centrifugation to remove smaller Aβ(1–42) assembly states. Fibrils are illustrated in (A) and shows the height image of the surface. Image (A) was used to determine a height profile (B) of the surface indicated by the black bar in (A).
(PNG)

Figure S9 **Development of the baseline after immobilization of: A) ∼150 RU N-terminally biotinylated Aβ(1–42) monomers, B) ∼200 RU Aβ(1–42) oligomers, C) ∼400 RU Aβ(1–42) fibrils.** Aβ(1–42) oligomers and fibrils were composed of a 1∶10 ratio of amino-terminally biotinylated Aβ(1–42) and non-biotinylated Aβ(1–42).
(PNG)
