# Supplementary figures and images for: Immobilization of Homogeneous Monomeric, Oligomeric and Fibrillar Aβ Species for Reliable SPR Measurements
Source: PLoS One. 2014 Mar 3;9(3):e89490. doi: 10.1371/journal.pone.0089490 (PMC3940443; doi:10.1371/journal.pone.0089490)

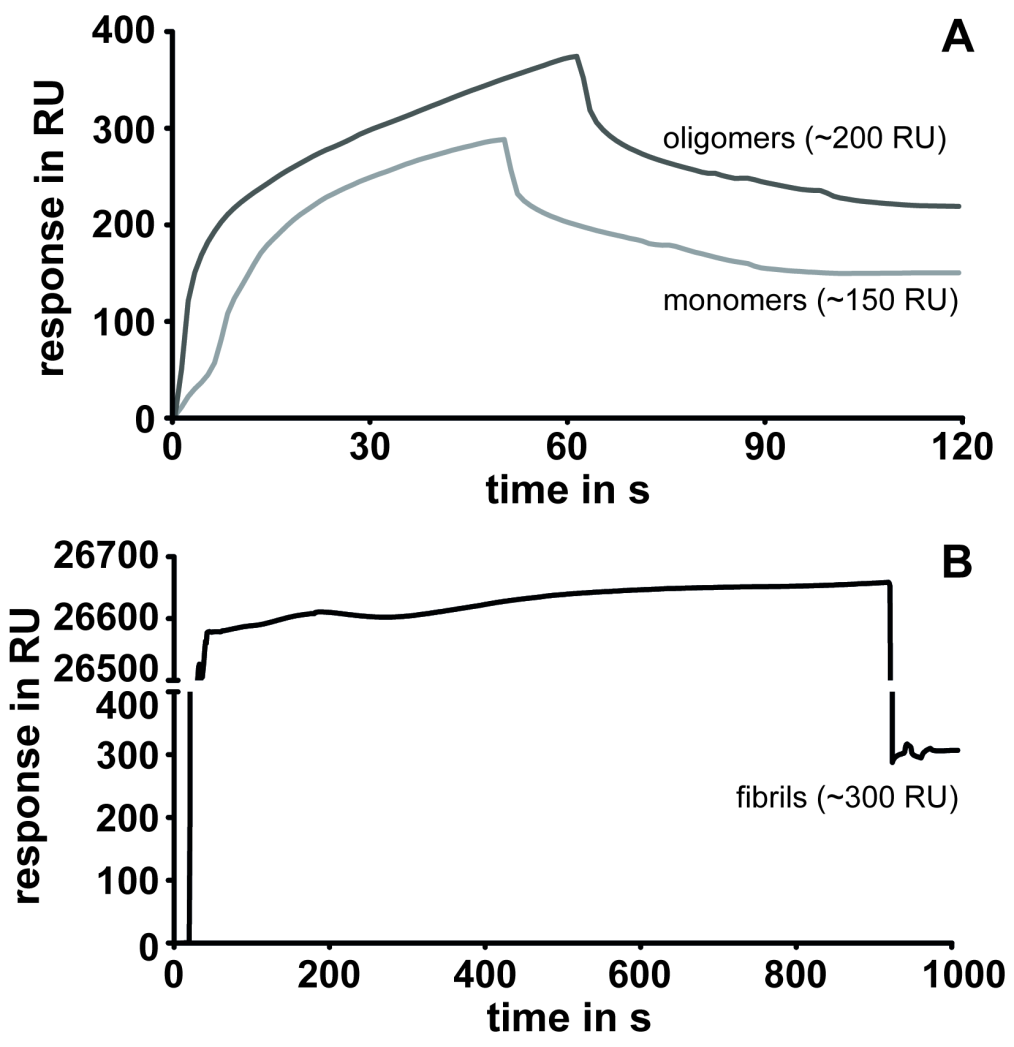

Supplement: Figure S1 — Overlay of sensorgrams obtained during immobilization of three different Aβ(1–42) assembly states on a streptavidin sensor chip. Oligomers and fibrils were prepared in a 1∶10 molar ratio of amino-terminally biotinylated and non-biotinylated Aβ(1–42), whereas monomers were completely biotinylated at the carboxy-termini. Final immobilized amounts are given in brackets. Shown are examples of sensorgrams obtained during immobilization of Aβ(1–42) monomers and oligomers (A) as well as fibrils (B). Because the procedure involves changes in buffer, these sensorgrams don't allow conclusions about association and dissociation rates of the immobilized Aβ(1–42) assembly states. After a few hours a stable baseline decay dependent on the immobilized assembly state was reached. RU: response units. (PNG) [file pone.0089490.s001.png]

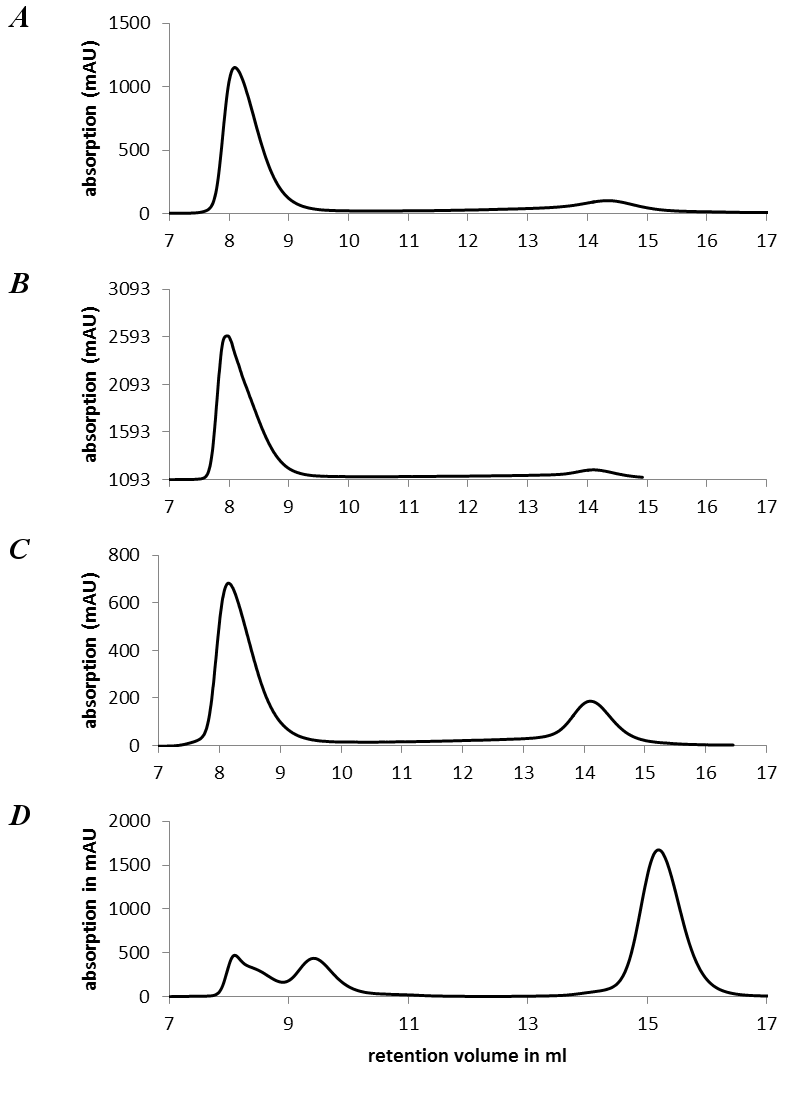

Supplement: Figure S2 — Size exclusion chromatography profile at 214 nm of A) 100% C-terminal biotinylated Aβ(1–42), B) 100% N-terminal biotinylated Aβ(1–42), C) 10% N-biotinylated Aβ(1–42)/90% Aβ(1–42), D) Molecular weight standard with Aprotinin (6.5 kDa), Lysozyme (14.4 kDa) and Conalbumin, Catalase, Aldolase, Ferritin in the void volume with Superdex 75 10/300 GL. Oligomers elute partly within the void volume and the monomers at ∼9 kDa. AU: absorption units at 214 nm. (PNG) [file pone.0089490.s002.png]

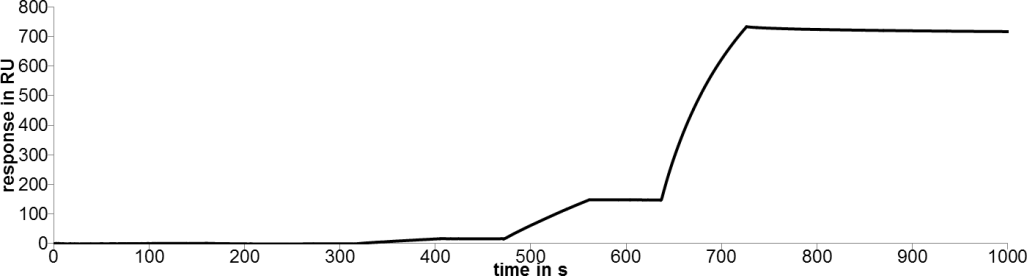

Supplement: Figure S3 — SPR sensorgram depicting binding of monoclonal IgG antibody 6E10 to N-terminally biotinylated Aβ(1–42) monomers immobilized on a streptavidin-coated SPR sensor chip. (PNG) [file pone.0089490.s003.png]

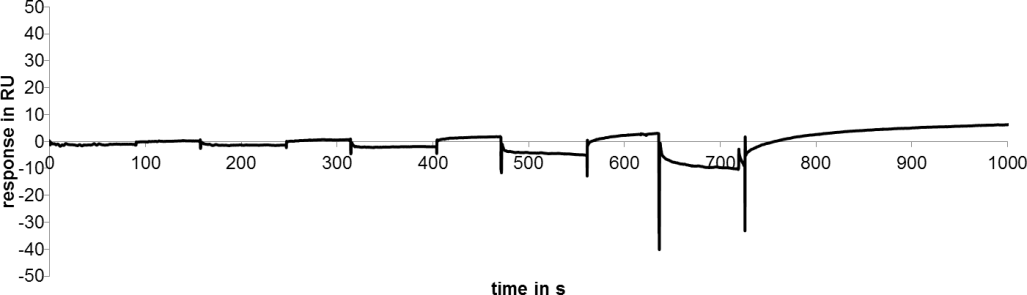

Supplement: Figure S4 — SPR sensorgrams depicting binding pattern of scFv IC16 to ∼1200 RU N-terminally biotinylated Aβ(1–42) monomers, immobilized on a streptavidin-coated SPR sensor chip. (PNG) [file pone.0089490.s004.png]

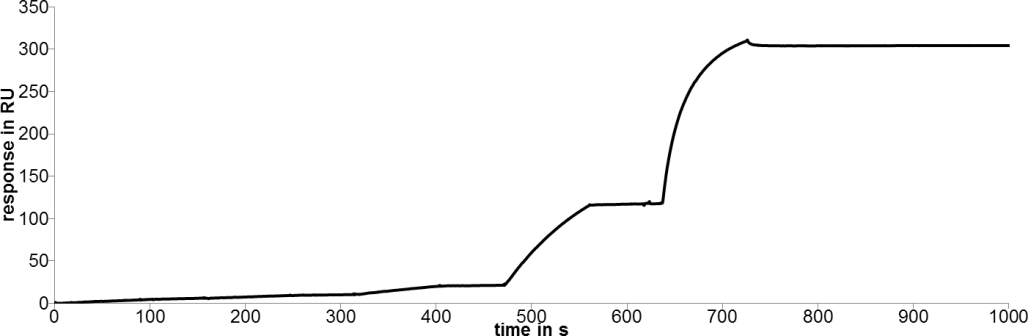

Supplement: Figure S5 — SPR sensorgram depicting binding of monoclonal IgG antibody 6E10 to C-terminally biotinylated Aβ(1–42) monomers immobilized on a streptavidin-coated SPR sensor chip. (PNG) [file pone.0089490.s005.png]

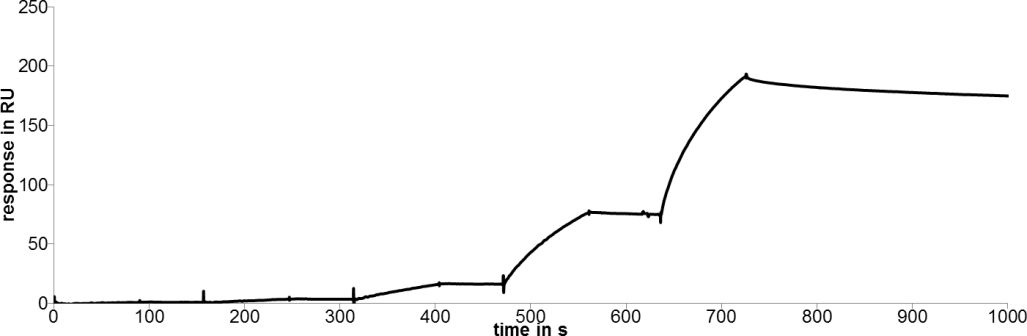

Supplement: Figure S6 — SPR sensorgram depicting binding of monoclonal IgG antibody 6E10 to Aβ(1–42) oligomers immobilized on a streptavidin-coated SPR sensor chip. Aβ oligomers were composed of a 1∶10 ratio of amino-terminally biotinylated Aβ(1–42) and non-biotinylated Aβ(1–42). (PNG) [file pone.0089490.s006.png]

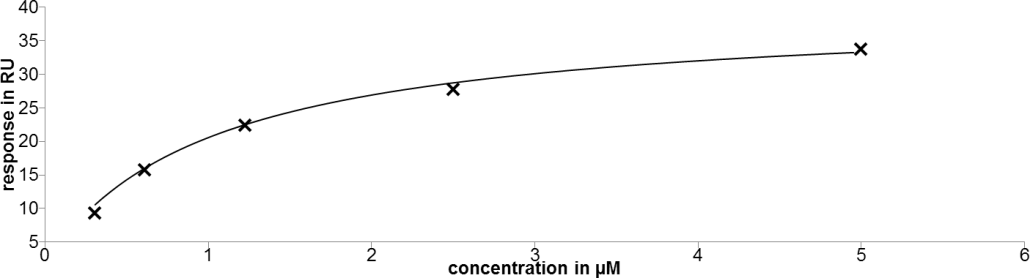

Supplement: Figure S7 — Steady-state analysis of scFv-IC16 binding to immobilized C-terminally biotinylated Aβ(1–42) monomers. The dissociation constant K D for a 1∶1 interaction is calculated from equation Req = (C*Rmax)/(K D+C), where C refers to the analyte concentration, Req to the obtained equilibrium binding levels and Rmax to the maximum analyte binding capacity of the surface. Values for K D and Rmax were determined to 0.97 µM and 39.7 RU with a corresponding χ2 value of 0.38. (PNG) [file pone.0089490.s007.png]

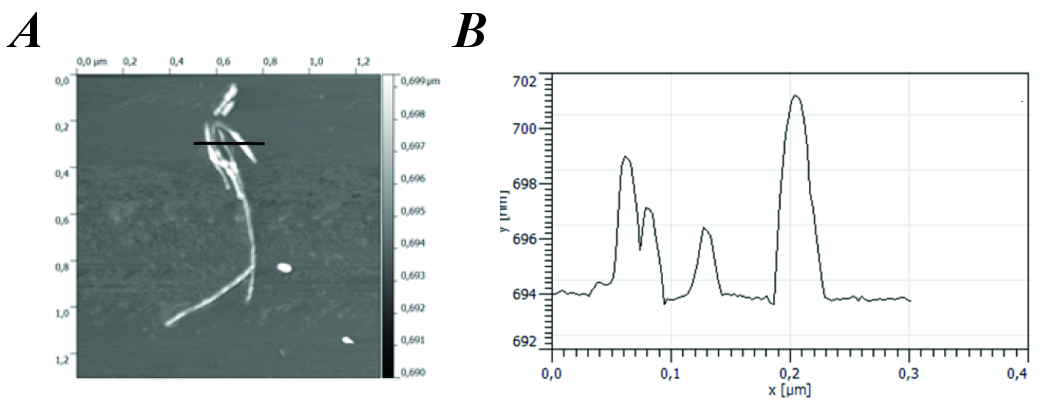

Supplement: Figure S8 — Analysis of Aβ(1–42) fibrils after density gradient centrifugation (DGC) by atomic force microscopy. The fibrils were created in 10 mM sodium phosphate buffer (pH 7.4) and separated by density gradient centrifugation to remove smaller Aβ(1–42) assembly states. Fibrils are illustrated in (A) and shows the height image of the surface. Image (A) was used to determine a height profile (B) of the surface indicated by the black bar in (A). (PNG) [file pone.0089490.s008.png]

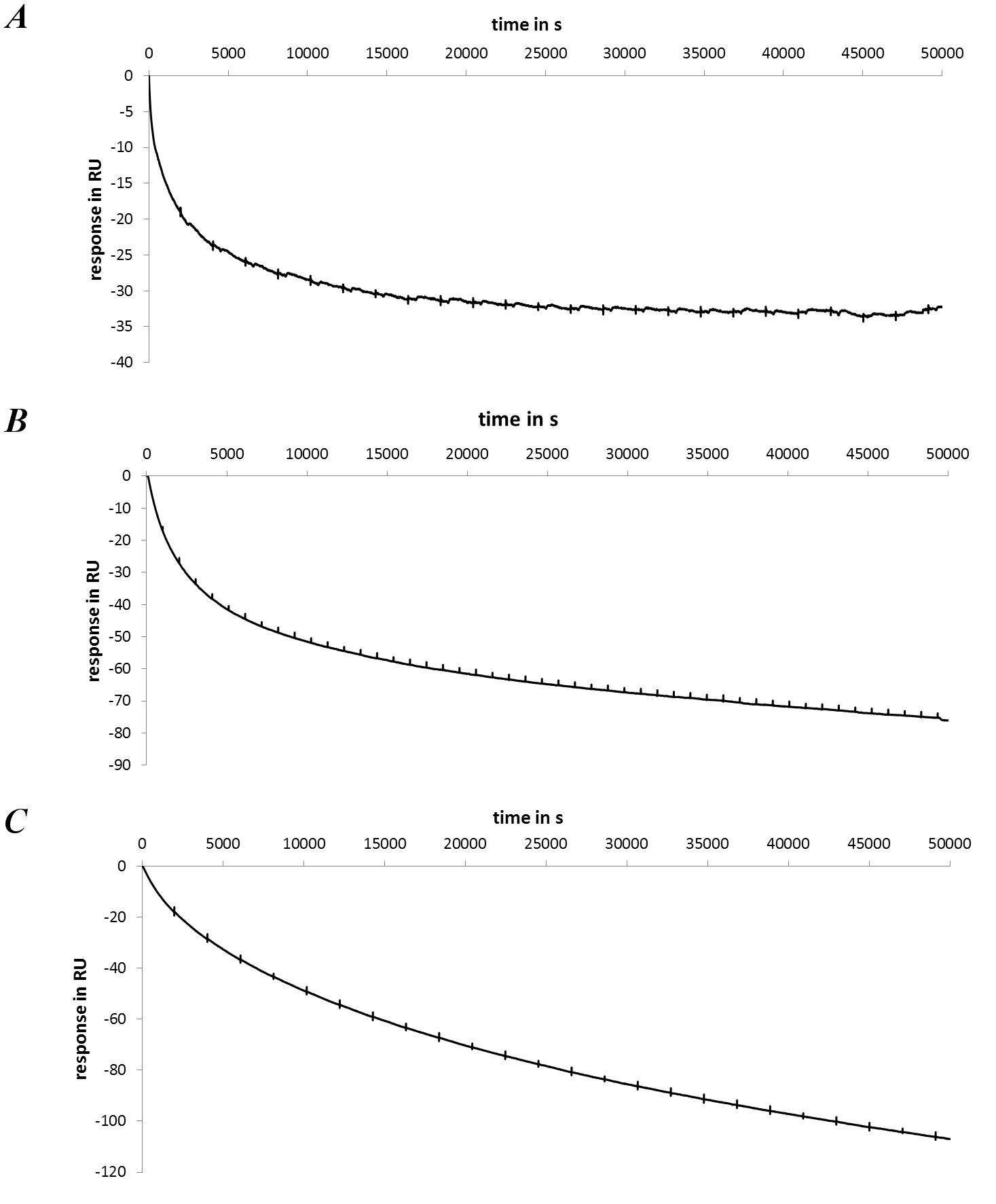

Supplement: Figure S9 — Development of the baseline after immobilization of: A) ∼150 RU N-terminally biotinylated Aβ(1–42) monomers, B) ∼200 RU Aβ(1–42) oligomers, C) ∼400 RU Aβ(1–42) fibrils. Aβ(1–42) oligomers and fibrils were composed of a 1∶10 ratio of amino-terminally biotinylated Aβ(1–42) and non-biotinylated Aβ(1–42). (PNG) [file pone.0089490.s009.png]
